# Effectiveness of gonadotropin-releasing hormone agonists for ovarian function suppression in premenopausal patients with hormone receptor-positive breast cancer: a retrospective single-center real-world study

**DOI:** 10.1007/s10549-024-07323-9

**Published:** 2024-05-06

**Authors:** Yifei Chen, Ruyan Zhang, Ying Yan, Huiping Li, Guohong Song

**Affiliations:** Key Laboratory of Carcinogenesis and Translational Research (Ministry of Education/Beijing), Department of Breast Oncology, Peking University Cancer Hospital & Institute, Beijing, 100142 China

**Keywords:** Breast cancer, Ovarian function suppression, Gonadotropin releasing hormone agonists (GnRHa), Real-world

## Abstract

**Purpose:**

This study evaluated the effectiveness of ovarian function suppression (OFS) of various gonadotropin-releasing hormone agonists (GnRHa) combined with aromatase inhibitors (AI) in premenopausal patients with hormone receptor-positive (HR-positive) breast cancer. Potential risk factors associated with insufficient OFS were analyzed.

**Patients and methods:**

Premenopausal HR-positive breast cancer patients who had received AI with GnRHa were studied retrospectively. Patients were divided into different groups according to monthly or trimonthly GnRHa schedules they received, and the effectiveness of OFS was compared between groups. Insufficient OFS was defined as at least one instance of estradiol ≥ 30 pg/ml. Patient data was gathered from medical records for this comparison.

**Results:**

Of the 264 patients enrolled in this study, 117 were administered 3.6 mg of goserelin monthly (goserelin 1 M group), 63 received 3.75 mg of leuprorelin monthly (leuprorelin 1 M group) and 84 were given 11.25 mg of leuprorelin every three months (leuprorelin 3 M group). Overall, 7.20% experienced insufficient OFS. The incidence rates in the three GnRHa depot groups were 7.69%, 6.35%, and 7.14%, respectively, without a significant statistical difference (*P* = 0.900). Notably, younger patients exhibited a higher likelihood of insufficient OFS [OR = 0.900, 95%CI (0.824–0.982), *P* = 0.018].

**Conclusion:**

Insufficient OFS remains a concern during GnRHa and AI treatment. The effectiveness of the three GnRHa depots commonly used in China seems comparable. Younger patients face a heightened risk of insufficient OFS.

## Introduction

Hormone receptor-positive (HR-positive) breast cancer, accounting for 50–75% of all breast cancers, is the most prevalent subtype [1, 2]. Studies indicate that luminal A breast cancer, which requires endocrine therapy, is more common in East Asian women under 50 [3]. In China, breast cancer diagnosis often occurs at a younger age compared to Western countries, with about 60% of patients remaining premenopausal. A 2017 study revealed that 16.4% of new breast cancer cases were diagnosed in women under 40 [4].

With the continuous update of the follow-up results of The Suppression of Ovarian Function Trial (SOFT) and the Tamoxifen and Exemestane Trial (TEXT) studies [5–7], ovarian function suppression (OFS) is recommended in conjunction with adjuvant endocrine therapy for high-risk patients [8, 9]. For advanced HR-positive breast cancer in premenopausal women, OFS is essential to induce a postmenopausal state, especially when using targeted medications like cyclin-dependent kinase 4/6 (CDK4/6) inhibitors alongside endocrine therapy. Studies [10, 11] suggest that both gonadotropin-releasing hormone agonist**s** (GnRHa) and oophorectomy are effective in achieving this. GnRHa, preferred for its non-invasiveness and reversibility, is the most common OFS method.

However, the SOFT Estrogen Substudy (SOFT-EST) and other studies have highlighted that a significant number of patients experience inadequate OFS with GnRHa [12–14]. This underscores the need to examine the OFS effectiveness of GnRHa. This retrospective study aims to evaluate the real-world frequency of insufficient OFS and associated risk factors in premenopausal breast cancer patients using GnRHa, which pilots the understanding of GnRHa ovarian suppression efficacy in the Asian population. The clinical characteristics of these patients have also been described in our study.

## Materials and methods

### Study design and patients

We reviewed a database from the Department of Breast Oncology at Peking University Cancer Hospital, spanning January 2009 to March 2021. This study focused on premenopausal HR-positive breast cancer patients. Premenopausal status was defined according to the National Comprehensive Cancer Network (NCCN) guidelines, excluding those meeting any criteria for menopause [8]. NCCN menopause criteria: (1) prior bilateral oophorectomy, (2) age ≥ 60 years, (3) age < 60 with amenorrhea for ≥ 12 months in the absence of prior chemotherapy, receipt of tamoxifen, toremifene, or ovarian suppression and estradiol (E2) and follicle-stimulating hormone (FSH) in the post-menopausal range, (4) age < 60 years: chemotherapy-induced amenorrhea for ≥ 12 months with E2 and FSH in post-menopausal range on serial assessments; (5) age < 60 years: on tamoxifen with E2 and FSH level in post-menopausal range.

In this study, the database was meticulously searched for patients who had been prescribed GnRHa and had E2 levels measured. The study included patients who received GnRHa combined with aromatase inhibitor (AI) therapy, with or without targeted therapy. Exclusions were made for patients undergoing concurrent chemotherapy or using GnRHa for ovarian protection during chemotherapy. Serum E2 levels were measured 3–6 months within 1 year after starting endocrine treatment, with insufficient OFS defined as at least one occurrence of E2 ≥ 30 pg/ml. Throughout this period, patients consistently received the same GnRHa depots determined by their physicians, and there was no systematic variation in hormone measurement interval strategies. This ensured that the E2 tests analyzed were specifically conducted during the period when patients were receiving both GnRHa and AI treatment, thereby focusing on a very specific patient group for this research.

Clinical characteristics and hormone levels were extracted from outpatient and inpatient records. These included age and Body Mass Index (BMI) at the start of GnRHa therapy, type of GnRHa and endocrine therapy used, previous chemotherapy, and disease stage. Patients were categorized into age groups using 40 years as a cutoff, based on the European School of Oncology (ESO)-European Society of Medical Oncology (ESMO) fifth international consensus guidelines for breast cancer in young women (BCY5) guidelines [15]. The disease stage was classified according to the American Joint Committee on Cancer (AJCC) staging system, with advanced breast cancer when distant metastases were present, otherwise as early-stage breast cancer.

This retrospective single-center real-world study received approval from the Ethics Committee of Peking University Cancer Hospital (Approval ID: 2021YJZ47).

### Study objectives

The primary objective of this study is to ascertain the percentage of patients experiencing at least one occurrence of E2 ≥ 30 pg/ml with GnRHa treatment. The secondary objectives include comparing the effectiveness of OFS across different GnRHa dosing groups and identifying potential risk factors for insufficient OFS.

### Sample management and hormone assays

Serum E2 levels were measured using an electrochemiluminescence immunoassay (ECLIA) on Roche Cobas 8000 E602 immunology analyzer in Peking University Cancer Hospital.

### Statistical methods

All analyses were performed with SPSS version 25. The Mann–Whitney test is employed for the comparing groups when the data did not follow a normal distribution. Count data were presented as case numbers and percentages (%), and group comparisons were made using the chi-square (*χ*^2^) test or Fisher exact test. Binary logistic regression analysis was applied to explore the correlation between insufficient ovarian suppression and factors like BMI, age, prior chemotherapy, and clinical stage. The deletion method was employed for handling missing data. Statistical significance was set at a *P* value < 0.05 (two-sided).

## Results

### Patient characteristics

This retrospective study included 264 patients undergoing GnRHa combined with AI therapy. The patient baseline characteristics are detailed in Table 1. The cohort comprised 134 patients with early-stage breast cancer (EBC) and 130 with advanced breast cancer (ABC). A significant majority, 89.39%, had undergone previous chemotherapy, with 70.83% receiving either cyclophosphamide or carboplatin. The median age at the start of GnRHa therapy was 43.59 years (range 25.47–57.25 years), with 90 patients (34.09%) aged 40 or younger and 174 patients (65.91%) elder than 40. The median BMI was 23.53 kg/m^2^ (range 14.98–36.72 kg/m^2^), though 18 patients lacked BMI records due to missing height and body weight information. Among the 130 ABC patients, 72 received chemotherapy as first line treatment in a metastatic setting, and 50 underwent endocrine ± target therapy after previous adjuvant chemotherapy. Notably, 8 patients received CDK4/6 inhibitors and 3 patients had everolimus in the advanced setting.

**Table 1:** Baseline characteristics in different GnRHa groups

	Total	GnRHa			*P* value
		Goserelin 1 M *n* = 117	Leuprolide 1 M *n* = 63	Leuprolide 3 M *n* = 84	
Age (years)					0.449
≤ 40, *n* (%)	90 (34.09%)	36 (30.77%)	21 (33.33%)	33 (39.29%)	
> 40, *n* (%)	174 (65.91%)	81 (69.23%)	42 (66.67%)	51 (60.71%)	
Median age (years, range)	43.59 (25.47–57.25)	44.05 (25.47–57.25)	43.3 (30.02–56.52)	42.7 (29.72–56.94)	0.114
Median BMI (kg/m^2^, range)	23.53 (14.98–36.72)	23.91 (18.07–31.55)	23.19 (14.98–36.46)	23.59 (17.63–36.72)	0.882
Disease stage					0.054
EBC, *n* (%)	134 (50.76%)	69 (58.97%)	29 (46.03%)	36 (42.85%)	
ABC, *n* (%)	130 (49.24%)	48 (41.03%)	34 (53.97%)	48 (57.15%)	
Previous chemotherapy					0.009
Yes, *n* (%)	236 (89.39%)	97 (82.91%)	59 (93.65%)	80 (95.24%)	
No, *n* (%)	28 (10.61%)	20 (17.09%)	4 (6.35%)	4 (4.76%)	
Previous chemo agent containing either CTX or carboplatin					0.764
Yes, * n* (%)	187 (70.83%)	80(68.38%)	47 (74.60%)	60 (71.43%)	
No, * n* (%)	77 (29.17%)	37 (31.62%)	16 (25.40)	24 (28.57%)	

*GnRHa* gonadotrophin-releasing hormone agonists, *BMI* body mass index, *EBC* early-stage breast cancer, *ABC* advanced breast cancer, *CTX* cyclophosphamide

Patients were treated with one of three GnRHa depots: goserelin 3.6 mg monthly (Goserelin 1 M), leuprorelin acetate 3.75 mg monthly (Leuprorelin 1 M), or leuprorelin acetate 11.25 mg every 3 months (Leuprorelin 3 M), with 117, 63, and 84 patients in each group, respectively. No significant differences were observed in median age, BMI, disease stages, or history of cyclophosphamide or carboplatin treatment across these groups. However, a smaller proportion (82.91%) in the Goserelin 1 M group had prior chemotherapy exposure.

Characteristics of patients with sufficient versus insufficient OFS are summarized in Table 2. There were no significant differences in median age, BMI, disease stages, previous chemotherapy, or GnRHa depots between the two groups. However, patients in the insufficient suppression group were notably younger (≤ 40 years).

**Table 2:** Baseline characteristics by ovarian function suppression status

	Total *n* = 264	insufficient OFS *n* = 19	Sufficient OFS *n* = 245	*P*-value
Age (years)				0.023
≤ 40, * n* (%)	90(34.09%)	11(57.89%)	79(32.24%)	
> 40, * n* (%)	174(65.91%)	8(42.11%)	118(67.76%)	
Median age (years, range)	43.59 (25.47–57.25)	38.17 (30.02–53.50)	43.66 (25.47–57.25)	0.067
Median BMI (kg/m^2^, range)	23.53 (14.98–36.72)	25.48 (14.98–36.46)	23.44 (17.63–36.72)	0.234
Disease stage				0.208
EBC, * n* (%)	134(50.76%)	7(36.84%)	127(51.84%)	
ABC, * n* (%)	130(49.24%)	12(63.16%)	118(48.16%)	
Previous chemotherapy (*n*, %)				0.432
Yes, * n* (%)	236(89.39%)	18(94.74%)	218(88.98%)	
No, * n* (%)	28(10.61%)	1(5.26%)	27(11.02%)	
Previous chemo agent containing either CTX or carboplatin				0.810
Yes, * n* (%)	187 (70.83%)	13 (68.42%)	174 (71.02%)	
No, * n* (%)	77 (29.17%)	6 (31.58%)	71 (28.98%)	
GnRHa				0.900
Goserelin 1 M, * n* (%)	117 (44.32%)	9 (47.37%)	108 (44.08%)	
Leuprorelin 1 M, * n* (%)	63 (23.86%)	4 (21.05%)	59 (24.08%)	
Leuprorelin 3 M, * n* (%)	84 (31.82%)	6 (31.58%)	78 (31.84%)	

*OFS* ovarian function suppression, *GnRHa* gonadotrophin-releasing hormone agonists, *BMI* body mass index, *EBC* early-stage breast cancer, *ABC* advanced breast cancer, *CTX* cyclophosphamide

### Insufficient OFS across groups

Insufficient OFS, defined as at least one instance of E2 ≥ 30 pg/ml during GnRHa treatment, was observed in 19 of the 264 patients (7.2%). The rates of insufficiency were 7.69% (9/117) in Goserelin 1 M, 6.35% (4/63) in Leuprorelin 1 M, and 7.14% (6/84) in Leuprorelin 3 M, with no significant differences (*P* = 0.900). In patients younger and older than 40, the rates were 12.22% (11/90) and 4.60% (8/174), respectively.

### Risk factors for insufficient OFS

Multivariable binary logistic regression analysis identified younger age as a potential risk factor for insufficient OFS [OR = 0.900, 95%CI (0.824–0.982), *P* = 0.018]. BMI, previous experience with chemotherapy and disease stage did not significantly impact the OFS effectiveness (Table 3).

**Table 3:** Multivariable logistic regression results for risk factors of insufficient ovarian function suppression

Risk factor	OR	95%CI	*P*-value
Age	0.900	0.824–0.982	0.018
BMI	1.123	0.989–1.276	0.075
Previous chemotherapy	1.119	0.128–9.744	0.919
Disease stage	0.390	0.131–1.160	0.090

*OR* Odds ratio, *CI* Confidence interval, *BMI* Body Mass Index

## Discussion

This retrospective study is the first of its kind in China to address the issue of insufficient OFS. We found that 7.20% of premenopausal patients did not achieve adequate suppression, with younger age being the only significant risk factor identified. No statistically significant difference was found among 3 different GnRHa depots, including the 3-month schedule leuprorelin. Despite the growing focus on monitoring sex hormone levels during ovarian suppression therapy, few studies have investigated this issue (Table 4). The SOFT-EST substudy [12] reported the E2 levels of patients receiving GnRHa combined with exemestane as adjuvant endocrine therapy. The SOFT-EST substudy and its final analysis [12, 16] reported varying E2 levels in patients using GnRHa with exemestane, highlighting the complexity of estrogen suppression in this context. The hormone assays in the SOFT-EST substudy were performed at 3, 6, and 12 months, revealing a considerable number of patients with E2 levels above 2.72 pg/ml, 10 pg/ml, and 20 pg/ml. For the 4-year analysis, a significant proportion of patients had suboptimal estrogen suppression (SES), defined as two or more postbaseline E2 readings above 2.72 pg/ml, 10 pg/ml, 20 pg/ml, or vaginal bleeding. There were 2 real-world studies that reported the OFS efficacy of GnRHa combined with endocrine therapy. Burns et al. [14] evaluated the persistence of ovarian escape (OE) in patients receiving GnRHa plus adjuvant endocrine therapy, defining OE as E2 > 2.7 pg/ml (or E2 > 21 pg/ml if on tamoxifen) during AI therapy. Their study reported 23.9% of patients had OE at 3 months, and 6.5% of patients remained in OE during the 1-year test.Van Houdt et al. [13] conducted a retrospective monocentric study focusing on ovarian function recovery (OFR) in patients receiving AI ± GnRHa with a defined threshold for E2 levels. 3 of 48(6.3%) patients on AI + GnRHa had OFR.

**Table 4:** Studies of ovarian function suppression effectiveness

	SOFT-EST [13]	SOFT-EST substudy final analysis [24]	Burns [15]	Van Houdt [14]	This study
Disease stage	EBC	EBC	EBC	EBC	EBC + ABC
Patient number	86	83	46	48	264
GnRHa depot	Triptorelin 3.75 mg	Triptorelin 3.75 mg	Goserelin 3.6 mg Goserelin 10.8 mg Leuprorelin 3.75 mg Leuprorelin 11.25 mg	Triptorelin 3.75 mg Goserelin 3.6 mg	Goserelin 3.6 mg Leuprorelin 3.75 mg Leuprorelin 11.25 mg
Endocrine medication	AI	AI	AI + SERM	AI	AI
Hormone assay	GC/MS/MS	GC/MS/MS	LC–MS/MS	LC–MS/MS	ECLIA
E2 cutoff(pg/ml)	2.72/10/20	> 2.72/10/20 in ≥ 2 post-baseline samples	If on AI: > 2.7 If on SERM:21	10	30
Risk factor	Lower baseline FSH and LH	Lower FSH and LH, higher E2 at baseline	Age	Age < 50 years, Previous use of chemotherapy	Age ≤ 40 years
Insufficient OFS rate	34.2%, 17.7%, 12.7%	25%, 13%, 7%	6.5–23.9%	6.3%	7.20%

*SOFT-EST* the Suppression of Ovarian Function Trial Estrogen Substudy, *EBC* early-stage breast cancer, *ABC* advanced breast cancer, *GnRHa* gonadotrophin-releasing hormone agonists, *AI* aromatase inhibitor, *SERM* selective estrogen receptor modulator, *GC/MS/MS* gas chromatography-tandem mass spectrometry, *LC–MS/MS* liquid chromatography-tandem mass spectrometry, *ELCIA* electrochemiluminescence immunoassay, *OFS* ovarian function suppression, *E2* estradiol, *FSH* follicle-stimulating hormone, *LH* luteinizing hormone

Our study identified younger age as a key risk factor for insufficient OFS, with a significant association (*P* = 0.018). This finding aligns with previous research: Burns and Van Houdt [13, 14] reported that younger patients are more prone to ovarian escape or ovarian function recovery (*P* = 0.04, *P* = 0.024 respectively). Van Houdt’s study [13] further revealed that prior chemotherapy decreased the likelihood of OFR (*P* = 0.03). Moreover, the SOFT-EST substudy [12] provided additional context by showing that patients with at least one E2 > 2.72 pg/ml had lower baseline FSH(*P* = 0.002) and lower baseline luteinizing hormone (LH) (*P* = 0.004) than those with lower E2 levels. The SOFT-EST substudy final analysis [16] showed higher E2, lower FSH, and lower LH values were related to SES (*P* = 0.02, *P* < 0.01, *P* < 0.01 respectively). Due to the retrospective nature of this study, we did not have adequate FSH/LH measurements for evaluation. Our data did not show a significant association between previous chemotherapy or chemotherapy involving cyclophosphamide or carboplatin and insufficient OFS. However, it's important to consider that among the 122 advanced breast cancer (ABC) patients who underwent chemotherapy, 50 only received it in an adjuvant setting. This subset of patients was more likely to have regained ovarian function, potentially diluting the overall impact of chemotherapy on OFS efficacy.

In our study, three different GnRHa formulations were used, with the rates of insufficient OFS being 7.69% for Goserelin 1 M, 6.35% for Leuprorelin 1 M, and 7.14% for Leuprorelin 3 M, showing no significant differences among them. This finding is particularly relevant in the context of a study from the United States [17], which addressed challenges in adherence to GnRHa in the real-world adjuvant settings (more than one-third (37%) of patients were non-adherent to GnRHa), and 42% of patients had surgical ovarian ablation. Despite more extensive evidence supporting 1-month GnRHa regimens [7, 18, 19], recent research has shown that the OFS effectiveness and therapeutic outcomes are comparable between 1-month and longer-acting GnRHa formulations [20–23]. These position the 3-month depot GnRHa as a viable alternative for premenopausal patients with HR-positive breast cancer. Additionally, the approval of 6-month GnRHa formulations offers more convenience and choice in treatment schedules [24]. Given the evolving nature of breast cancer treatment, which increasingly mirrors the management of chronic conditions, it's crucial for clinicians to consider not just the clinical efficacy but also the quality of life and economic implications of treatment options, particularly in the face of global health challenges like the Covid-19 pandemic.

Given the situation of growing GnRHa patient populations, establishing a specific E2 level for monitoring ovarian suppression efficacy remains a challenge. Our study adopted the consensus in China, defining insufficient OFS as E2 ≥ 30 pg/ml. When comparing monthly and 3-monthly depots GnRHa hormone suppression efficacy, Kendzierski et al. [20] defined E2 < 40 pg/ml as ovarian ablation, while Masuda et al. and Kurebayashi et al. [21, 25] chose E2 < 30 pg/ml as the postmenopausal cutoff level. The TABLE study [26] was a randomized phase III trial comparing 3-monthly depot GnRHa with chemotherapy, in which E2 < 30 pg/ml was also used as the menopausal threshold. Not coincidentally, Lee et al. and Wu et al. [27, 28] chose this criterion when studying the effectiveness of 3-monthly and 6-monthly GnRHa formulations combined with tamoxifen therapy, respectively. These differences in E2 cutoff levels for postmenopausal status, ranging from E2 < 30 pg/ml to E2 < 40 pg/ml, highlight the lack of a standardized approach in measuring and interpreting hormone suppression efficacy in different studies. For premenopausal patients receiving AI as endocrine therapy, the lack of clear evidence linking very low E2 levels to improved clinical outcomes leads to varied standards for ovarian suppression in different studies. Smith et al. [29], for example, considered AI appropriate when E2 < 10 pmol/L(2.72 pg/ml) with elevated gonadotropin levels. The SOFT-EST substudy [12] and other real-world studies [13, 14] used a range of E2 cutoff**s**. Furthermore, the approach to E2 level measurement is not uniform globally. While some studies, particularly in Western countries, employ advanced techniques like liquid chromatography-tandem mass spectrometry (LC–MS/MS) for their superior sensitivity and specificity [30], others, including our study, rely on immunoassays due to their practicality and lower cost in settings like China. Our hospital laboratory used ECLIA with a Roche Cobas 8000 E602 immunology analyzer, which is reflective of the common practice in most Chinese laboratories.

This study acknowledges several limitations. The retrospective nature of this study meant that it did not have access to comprehensive data typically found in prospective studies, such as baseline hormone levels and menstrual cycle information. The absence of a consensus on E2 control standards and the unclear link between OFS efficacy and prognosis meant routine E2 level monitoring wasn't standard, limiting regular blood sample collection. Despite a sizeable sample, it's relatively small for the broader endocrine therapy population. Generalization of the results requires caution. High patient mobility and outpatient-based endocrine treatment at our tertiary hospital meant many patients didn't have regular visits, possibly indicating lower compliance. Conversely, the requirement for E2 level measurements might have unintentionally selected patients with higher adherence. Additionally, patients who underwent hormone testing might have been those with doctors more concerned about insufficient suppression. The follow-up data in this study is inadequate to analyze the correlation between OFS effectiveness and patient prognosis. However, theoretical implications on recurrence risk in HR-positive breast cancer patients due to insufficient suppression suggest the need for regular hormone level assessment in high-risk patients. Future research might reveal new biomarkers for more effectively assessing ovarian function suppression. Identifying potential predictors of inadequate OFS could greatly aid clinicians in making more informed treatment decisions. Focusing on this area might lead to significant improvements in tailoring therapies to individual patient needs.

## Conclusion

In conclusion, as more patients become eligible for endocrine therapy combined with GnRHa, future prospective studies are needed to examine the relationship between ovarian suppression efficacy and prognosis, as well as to identify risk factors for insufficient suppression. Physicians should monitor perimenopausal symptoms in patients treated with GnRHa and AI, and consider sex hormone monitoring, such as E2 levels, when necessary. This approach will aid in optimizing treatment and potentially improve patient outcomes.

## Data Availability

All data and materials supporting the conclusions of this article are available in the tables, which are available to authorized users.
